# Pencil beam all-optical ultrasound imaging

**DOI:** 10.1364/BOE.7.003696

**Published:** 2016-08-26

**Authors:** Erwin J. Alles, Sacha Noimark, Edward Zhang, Paul C. Beard, Adrien E. Desjardins

**Affiliations:** 1 Department of Medical Physics and Biomedical Engineering, University College London, London WC1E 6BT, UK; 2 Materials Chemistry Research Centre, UCL Department of Chemistry, London WC1H 0AJ, UK

**Keywords:** (170.7170) Ultrasound, (110.5125) Photoacoustics, (110.2350) Fiber optics imaging, (060.2380) Fiber optics sources and detectors, (170.0110) Imaging systems

## Abstract

A miniature, directional fibre-optic acoustic source is presented that employs geometrical focussing to generate a nearly-collimated acoustic pencil beam. When paired with a fibre-optic acoustic detector, an all-optical ultrasound probe with an outer diameter of 2.5 mm is obtained that acquires a pulse-echo image line at each probe position without the need for image reconstruction. B-mode images can be acquired by translating the probe and concatenating the image lines, and artefacts resulting from probe positioning uncertainty are shown to be significantly lower than those observed for conventional synthetic aperture scanning of a non-directional acoustic source. The high image quality obtained for excised vascular tissue suggests that the all-optical ultrasound probe is ideally suited for *in vivo*, interventional applications.

## 1. Introduction

All-optical ultrasound imaging has recently been demonstrated to yield high-quality images that compare favourably to those obtained with conventional piezoelectric ultrasound probes [1–7]. In all-optical ultrasound probes, ultrasound is generated photoacoustically through pulsed or modulated illumination of an optically absorbing coating, where thermal deposition causes an increase in pressure that propagates through the surrounding medium as an acoustic wave [8]. Back-scattered acoustic waves are typically detected using Fabry-Pérot etalons [2,3,6,9,10] or ring resonators [5,11].

An optical acoustic source fabricated on the tip of an optical fibre can generate ultrasound with bandwidths and pressures comparable to or better than those generated by conventional piezoelectric transducers [12–14]. When paired with a fibre containing an optical acoustic detector, an all-optical acoustic probe is obtained that is readily miniaturised and inexpensive to fabricate. Such all-optical ultrasound probes, which typically comprise two optical fibres (one each for transmission and reception), are ideally suited to minimally invasive interventional applications where space is limited.

Images of *ex vivo* tissue have previously been acquired through precise motorised scanning of an all-optical ultrasound probe across a synthetic aperture, whilst recording pulse-echo signals at uniform intervals [7]. Due to the low directionality of the acoustic source and the high sensitivity of the detector, the resulting images exhibit high resolution and low noise levels. However, the paradigm of mechanically scanning a synthetic aperture is unrealistic in an interventional setting, where micron-scale accuracy in probe position manipulation or tracking is currently not possible. As will be shown below, the resulting positioning uncertainty can introduce strong artefacts upon image reconstruction.

In this study, a different approach is presented where a highly directional optical acoustic source is used to generate a nearly-collimated pencil beam. Only structures located within the pencil beam are insonified and generate a pulse-echo response, and hence only a single recording is required to obtain an image line. As no image reconstruction is required to focus the acoustic energy, the positioning uncertainty image artefacts can be mitigated. The performance of a non-directional and a directional probe are compared both in the absence and presence of positioning errors, and the image quality obtained with the directional probe is demonstrated on *ex vivo* vascular tissue.

## 2. Methods

Two all-optical pulse-echo ultrasound imaging probes were developed. The first probe was non-directional, where ultrasound was generated at the distal end of a flat-cleaved optical fibre. The second probe achieved an acoustic pencil beam through directional ultrasound transmission by a concave surface. With both probes, pulsed laser light with a duration of 2 ns and a wavelength of 1064 nm (SPOT-10-500-1064, Elforlight, U.K.) was delivered to an optically absorbing coating to generate ultrasound via the photoacoustic effect. To record the acoustic pulse-echo signals, a fibre-optic acoustic detector was used that comprised a Fabry-Pérot cavity at the distal end [10]. This detector was interrogated by measuring the detector’s reflectivity with a tunable laser (TUNICS T100S-HP, Yenista, France) and a custom photodiode. The wavelength of the interrogation laser was adjusted to correspond with the peak derivative of the detector’s cavity transfer function [15]. Acoustic data were sampled using a high-speed data acquisition card (250 MSa/s, 14-bit; M4i.4420-x8, Spectrum, Germany). Acoustical cross-talk between the optical source and detector was suppressed using the method described in [7], and digital time gain compensation was applied to compensate for geometrical attenuation.

### 2.1. Non-directional probe

In the non-directional probe, ultrasound was generated in an optically absorbing coating deposited on a step-index optical fibre (core/cladding diameter: 200/220 μm). This coating comprised a thin (≤ 1 μm) layer of functionalised carbon nanotubes as optical absorbers and a 20 μm thick layer of polydimethylsiloxane (PDMS) as an elastomeric host, and was dip-coated onto the distal end of the optical fibre [14].

### 2.2. Pencil beam probe

**Fig. 1:**
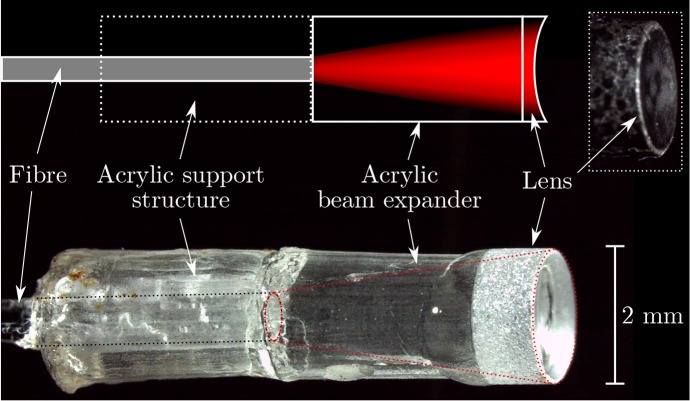
Schematic (top) and photograph (bottom) of the focussed optical ultrasound source. The inset (top right) shows a photograph of the probe after applying an optically absorbing coating.

In the directional probe, the geometrical focus of a spherical plano-concave microlens (diameter: 2 mm, radius of curvature: 3.4 mm; SLM-02-04N, OptoSigma, France) was employed to generate a nearly-collimated acoustic pencil beam. This lens was glued to an acrylic spacer (diameter: 2.0 mm, thickness: 3 mm) to expand the diverging beam emitted by a step-index optical fibre (core/cladding diameter: 600/630 μm, NA: 0.39) across the concave lens surface (Fig. 1). For mechanical support, a hollow acrylic cylinder (thickness: 3 mm) was glued around the fibre, and the assembly was enclosed by adhesive heat shrink for added mechanical protection. The concave lens surface was coated in black paint (Carbon Black Professional Spray Paint, Liquitex, OH, USA) to provide optical absorption for photoacoustic excitation.

The emitted acoustic field was measured by scanning a calibrated needle hydrophone (75 μm, Precision Acoustics, U.K.) across a 5 mm × 5 mm grid at a 50 μm step size using two orthogonal motorised stages (MTS50/M-Z8 + TDC001, Thorlabs, Germany). This hydrophone was positioned an axial distance of 3.0 mm from the acoustical source, and its signal was amplified by 20 dB using a high-bandwidth pre-amplifier (DHPVA-200, Femto, Germany). The pulse-echo signal recorded in the location corresponding to the highest recorded pressure was used to determine the acoustic pulse shape and bandwidth. The three-dimensional data set was subsequently propagated to different axial distances using the angular spectrum approach [16] to determine the full-width half maximum beam width at every distance.

### 2.3. Data acquisition and image reconstruction

To excite the pencil beam source, the laser was tuned to its maximum pulse energy of 42.0 μJ (corresponding to a fluence of 1.34 mJ/cm^2^), which occurred at a pulse repetition rate of 500 Hz, and pulse-echo signals were amplified by 20 dB and averaged over 100 recordings. The envelopes of the A-scans were directly displayed as image lines without reconstruction, and the spatial offset (2.5 mm) between the centres of the source and detector was accounted for in the conversion from time to axial distance. To excite the non-directional probe, the laser parameters were adjusted (pulse repetition rate: 100 Hz, pulse energy: 30.4 μJ, fluence: 96.8 mJ/cm^2^) to avoid thermal damage to the optical coating, and 10 recordings were averaged without pre-amplification. Images were reconstructed using the delay-and-sum algorithm [17], and the envelope of the resulting B-mode images was taken along the axial dimension.

### 2.4. Positioning uncertainty

The effect of positioning uncertainty on the image quality was studied, both numerically and experimentally, using a phantom consisting of a circular specular reflector (diameter: 240 μm). Experimentally, this target was realised by the tip of a graphite rod (diameter: 240 ± 10 μm). The target was positioned at various axial distances from the acoustical source in 1 mm intervals. This phantom was imaged (in simulation and experiment) by mechanically scanning either the non-directional or the pencil beam probe along a 5 mm long line at a step size of 25 μm, and recording pulse-echo signals at each location. In both numeric simulations and experiment, probe positioning uncertainty was introduced by deliberately adding positioning errors to each scan location in both the axial (depth) and lateral (scan dimension) directions. These positioning errors were sampled from uniform random distributions with ranges of 0 (no error), ±10, ±20, ±30, ±40 and ±50 μm. The positioning errors were identical for both probes. An additional experiment was performed where axial and lateral positioning errors that ranged between ±100 and ±2000 μm at 100 μm increments were introduced numerically through data resampling rather than experimentally through spatial offsets.

In numerical simulations, the pressure at the location of the disc target was computed using the FOCUS package [18]. For the non-directional probe, the source was modelled as a disc (diameter: 200 μm); for the directional probe, as a spherical section (radius of curvature: 3.4 mm). The diameter of the simulated lens was slightly smaller than that of the actual lens to account for an inhomogeneous illumination; its value (1.5 mm) was determined empirically by matching the beam diameter for axial depths up to 7 mm to that obtained from measured data. The target was modelled as a disk (diameter: 240 μm) of point scatterers spaced 2 μm apart, and the free-space Green’s function [19] was used to propagate the scattered waves back to the detector. The measured acoustic pulse shape was incorporated by means of temporal convolution.

### 2.5. Tissue imaging

To demonstrate the image quality achieved with the pencil beam probe, *ex vivo* images of an opened section of porcine aorta wall were acquired. Both a 1D line scan (30 mm long, 100 μm step size) across two side-branches and a 2D grid scan (7 mm × 10 mm, 50 μm step size) across a single side-branch were performed using motorised translation in the absence of deliberate positioning errors.

## 3. Results

### 3.1. Acoustic field measurements

**Fig. 2:**
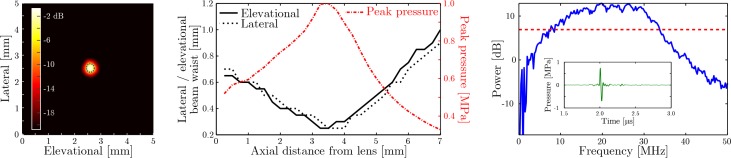
**Left:** maximum intensity of the transmitted acoustic field measured at an axial distance of 3 mm with the full-width half maximum contour indicated by the dotted blue curve. **Middle:** spatial extent of the acoustic beam in the elevational (black solid curve) and lateral direction (black dashed curve), together with the peak acoustic amplitude (red dash-dotted curve), as a function of axial depth. The acoustic data were measured at a distance of 3 mm and numerically propagated to the remaining depths. **Right:** power spectrum of the A-scan (shown in the inset) corresponding to the peak acoustic intensity measured at a distance of 3 mm. The dashed red line indicates the −6 dB level relative to peak power used to measure the bandwidth of the transmitted signal.

At an axial distance of 3 mm from the pencil beam source, the acoustic field is tightly confined (Fig. 2, left) to a circle with a diameter of approximately 0.3 mm. Propagating this acoustic field to different axial distances (Fig. 2, middle) reveals that the narrowest beam waist coincides with the geometrical focus of the coated lens (radius of curvature: 3.4 mm), that the beam exhibits circular symmetry around the axial axis, and that the beam diameter is narrower than 1 mm for axial distances up to 7 mm. As the ultrasound is not focused upon reception, the beam diameter is equivalent to the lateral resolution. The bi-polar pulse shape and large bandwidth (−6 dB relative to peak power between 8 and 33 MHz; Fig. 2, right) result in a high axial resolution of 75 μm. The peak acoustic amplitude ranges between 0.35 and 1.0 MPa for axial distances up to 7 mm, and is limited by the maximum pulse energy of the applied laser.

### 3.2. Phantom imaging - positioning uncertainty

**Fig. 3:**
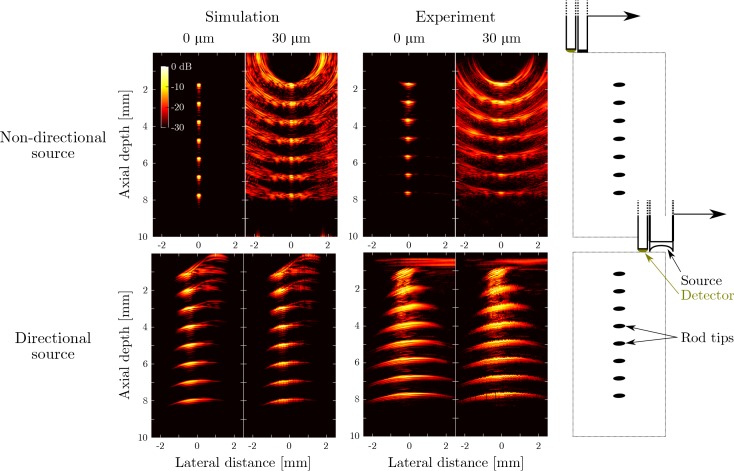
**Top row:** simulated (first two columns) and measured (middle two columns) all-optical ultrasound images obtained of a phantom using an unfocussed acoustic source. The phantom consisted of the tip of a single rod placed at different depths (right column); the resulting images are compounded into a single synthetic image. Simulations and experiments were performed both in the absence (“0 μm”) and presence (“30 μm”) of deliberate probe positioning errors. Positioning errors were applied to both the axial and the lateral axis and sampled from a uniform random distribution with a range of ±30 μm. **Bottom row:** the same panels are shown for the case where all-optical pulse-echo ultrasound data were acquired using the focussed acoustic source.

Both simulations and experiments confirm that the presence of modest positioning uncertainty (≤ 30 μm) introduces severe artefacts in images obtained with the non-directional probe (Fig. 3). While the actual object can still be recognised, the artefacts have nearly the same magnitude as the actual signal, and hence the image has strongly reduced contrast. Similar images obtained for the full set of tested positioning error ranges ( Visualization 1 and Visualization 2) reveal that significant artefact levels are introduced by positioning errors as small as ≤ 20 μm. By comparison, images obtained in both simulations and experiments using the pencil beam probe are insensitive to probe positioning errors of up to ±300 μm. While minor distortions can be observed in the presence of positioning uncertainty, no additional artefacts are introduced and hence the image contrast is unaffected.

Several differences between the simulated and experimental data are apparent. The artefacts observed in simulations for the directional probe (axial depths between 0 and 4 mm, lateral distances between 0 and 1.5 mm) can be attributed to an unfocussed edge wave generated by the outer ring of the coated lens. This edge wave and its corresponding artefact are less pronounced in the experimental case due to the absence of an optically absorbing coating at the sharp edge of the lens (Fig. 1). The horizontal artefact observed at an axial depth of around 0.5 mm is caused by incomplete suppression of the acoustic cross-talk between the source and receiver. In addition, slight differences in the lateral spatial resolution between simulated and experimental data were observed: for the directional probe, the lateral resolution obtained from experimental data was lower than than obtained from simulated data. This lower resolution may have arisen from spatial inhomogeneities in the excitation light distribution across the lens surface that were not accounted for in the simulation model.

### 3.3. Tissue imaging

**Fig. 4:**
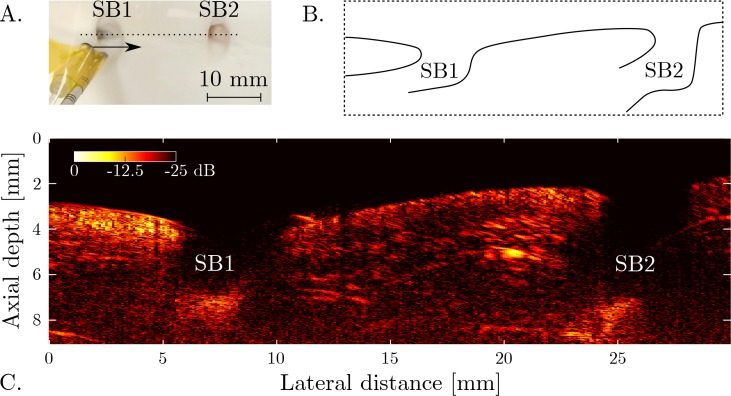
**A.** Photograph of the focussed probe positioned above an *ex vivo* aortic section containing two side-branches (SB1 and SB2). The probe was translated along the dotted line to scan a synthetic aperture. **B.** Schematic cross-section of the aorta wall and side-branches. **C.** All-optical ultrasound image acquired using the focussed acoustic source. The image, displayed on a logarithmic scale, was obtained without image reconstruction.

**Fig. 5:**
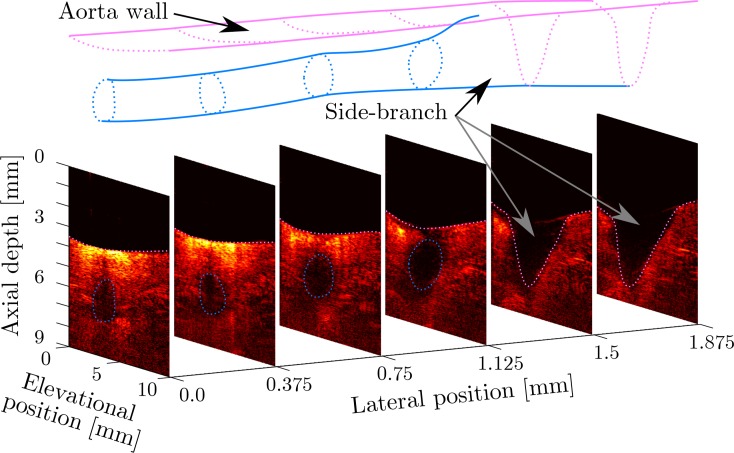
Cross-sections through a volumetric all-optical ultrasound image of an aorta section containing a side-branch (SB1 in Fig. 4). The geometry of the aorta wall and side-branch veering off to the left are indicated in purple and blue, respectively. All cross-sections are shown on the same logarithmic scale (40 dB dynamic range), and no image reconstruction was applied. The lateral axis was scaled to improve the visibility of the cross-sections.

By mechanically scanning the pencil beam probe across a line aperture, a 2D image of *ex vivo* aortic tissue (Fig. 4) was obtained. In this image, the aorta wall is clearly visible, and the shape and location of two bifurcations are readily observed. In addition, the geometry of two side-branches (SB1 and SB2) can be appreciated. The geometry of side-branch SB1 is more readily observed in the 3D image obtained by scanning the probe across a 2D grid (Fig. 5). A fly-through ( Visualization 3) and volumetric render ( Visualization 4) of this 3D image volume are provided as supplemental data.

## 4. Discussion and conclusion

In this work, a highly directional all-optical ultrasound probe was presented that employs geometrical focussing of the transmitted beam to form image lines without the need for image reconstruction. The presented directional probe was shown to generate a nearly collimated acoustic pencil beam that maintains a high pressure and bandwidth over a large axial distance, which was employed to image an *ex vivo* aorta containing side-branches in 2D and in 3D. To the authors’ knowledge, this is the first study in which imaging of biological tissue was performed with a geometrically focused optically transmitted acoustic beam.

Given its small outer diameter of 2.5 mm, the all-optical acoustic pencil beam probe is ideally suited to interventional and endoscopic applications of ultrasound imaging. These applications include endovascular imaging for guidance of aortic stent placement, where visualisation of side-branches is of crucial importance [20]. An all-optical acoustic pencil beam probe could be positioned within a steerable tip catheter that is manually or robotically scanned across a region of interest to build up an image. Using this approach in laparoscopic or fetoscopic contexts, an imaging aperture could be achieved that far exceeds the port size. For compatibility with other minimally invasive procedures, such as those performed in small-diameter vessels, the outer diameter could be further reduced by decreasing the lens diameter. However, this will decrease the tightness of the acoustical focus and consequently the lateral resolution. The spatial resolution could be improved through spatial deconvolution, particularly using implementations involving spatially varying kernels [21]. However, such approaches tend to be computationally intensive, and as such they would present significant challenges for real-time visualisation [22].

The spatial resolution with which the probe position could be controlled or measured using current optical and electromagnetic tracking systems (in the vicinity of several hundreds of micrometers [23,24]) exceeds the accuracy required for synthetic aperture focussing (approximately 20 μm). Consequently, using a non-directional probe in conjunction with synthetic aperture focussing gives rise to strong image artefacts and decreased image resolution and contrast. The presented directional probe does not rely on synthetic aperture focussing, and hence the image quality was shown to be virtually unaffected by the presence of positioning uncertainty of up to 300 μm; image contrast was maintained and only slight spatial distortions were observed that might be suppressed by applying spatial filtering. Thus, while the lateral resolution of the directional pencil beam probe was shown to be lower than what can be achieved through synthetic aperture scanning of a non-directional probe, the robustness of the image quality to randomised probe position uncertainty was strongly improved.

Fibre-optic acoustic technology has distinct advantages over the conventional piezoelectric alternative. Miniature acoustic sources can be deposited through dip- or spray-coating, thereby avoiding the micro-machining required to dice miniaturised piezoelectric transducers. An absence of electrical connections further simplifies transducer fabrication, and renders all-optical acoustic probes insensitive to electromagnetic interference. As a result, miniature optical acoustic probes can be manufactured inexpensively, are MRI compatible, and allow for simultaneous use of other imaging modalities. Additionally, optical ultrasound generation is non-resonant, and hence the source bandwidth and frequency can be tuned by varying the laser pulse duration or using modulated excitation [25]. Consequently, the same optically absorbing coating can be used to generate a wide bandwidth (for high resolution imaging), intermediate bandwidth (to improve penetration), or narrow bandwidth (for Doppler flow measurements or microbubble contrast).

In the experiments presented above, the peak acoustic pressure was limited by the pulse energy provided by the excitation laser; after prolonged use of the probe, no deterioration in acoustic performance of the sound-generating coating on the directional probe was observed. In particular, the cross-talk amplitude and wavefront monitored over a period of 4.5 hours varied by less than 3 %. However, preliminary experiments not reported here suggest the damage threshold fluence for the coating is up to 50 times higher than the fluence used in this work. Therefore, light sources with higher pulse energies could be employed to increase the peak acoustic pressure to tens of MPa, thereby enabling therapeutic use, as previously suggested by Baac *et al.* [12]. The peak acoustic amplitude could be further increased by switching to materials that convert optical to acoustic energy more efficiently, such as those based on multi-walled carbon nanotubes overcoated with PDMS [12,14].

Focussed or collimated acoustical beams for use in biomedical imaging have been generated through various approaches. For instance, an annular array of piezoelectric transducers (diameter: 25 mm) has been used to generate a non-diffracting Bessel beam [26]. Alternatively, miniature two-dimensional arrays (diameter: 2.5 mm) comprising either piezoelectric or capacitive micromachined ultrasound transducers (CMUTs) can be used to dynamically focus acoustic beams at different locations and depths [27]. Furthermore, single miniature geometrically focussed piezoelectric transducers (diameter: 5 mm) have previously been used in commercial ultrasound scanners [28], where images were generated by rapidly wobbling the transducer. While miniaturised piezoelectric or CMUT sources can yield bandwidths and pressures that are similar to those obtained with the all-optical probes presented in this study, their significantly higher manufacturing costs prohibit widespread interventional use where probes are treated as disposables. However, focussed piezoelectric or CMUT transducers can typically be used for both acoustic transmission and detection, and hence a better lateral resolution than demonstrated in this work can be obtained. Similarly, a more directional fibre-optic acoustic detector could be used to improve the lateral resolution. Alternatively, lenses with different diameters or radii of curvature could be used to manufacture acoustic sources that generate a tighter focus or a larger depth of field.

This study demonstrates how changing the geometry of the optical acoustic source enables a different imaging approach to all-optical ultrasound imaging that is robust to probe positioning uncertainty. With the resulting probe, it is expected that high quality images can be obtained *in vivo* using probe manipulation and tracking methods that are suitable to an interventional setting.
